# Methionine-producing tumor micro(be) environment fuels growth of solid tumors

**DOI:** 10.1007/s13402-023-00832-7

**Published:** 2023-06-15

**Authors:** Alexis A. Vega, Erin A. Marshall, Avery J. C. Noonan, Fernando Sergio Leitao Filho, Julia Yang, Greg L. Stewart, Fraser D. Johnson, Emily A. Vucic, Michelle E. Pewarchuk, Parag P. Shah, Brian F. Clem, Corey Nislow, Stephen Lam, William W. Lockwood, Steven J. Hallam, Janice M. Leung, Levi J. Beverly, Wan L. Lam

**Affiliations:** 1 Department of Biochemistry and Molecular Genetics, University of Louisville, Louisville, KY USA; 2 Brown Cancer Center, University of Louisville School of Medicine, 505 S. Hancock St. Rm 204, Louisville, KY 40202 USA; 3 Integrative Oncology, BC Cancer Research Centre, Vancouver, BC Canada; 4 Interdisciplinary Oncology Program, University of British Columbia, Vancouver, BC Canada; 5 Genome Science and Technology Program, University of British Columbia, Vancouver, BC Canada; 6 ECOSCOPE Training Program, University of British Columbia, Vancouver, BC Canada; 7 Centre for Heart Lung Innovation, St Paul’s Hospital, Vancouver, BC Canada; 8 NYU Langone Medical Center, New York, NY USA; 9 Faculty of Pharmaceutical Sciences, University of British Columbia, Vancouver, BC Canada; 10 Department of Pathology and Laboratory Medicine, University of British Columbia, Vancouver, BC Canada; 11 Department of Microbiology & Immunology, University of British Columbia, Vancouver, BC Canada; 12 Bioinformatics Program, University of British Columbia, Vancouver, BC Canada; 13 Biofactorial High-Throughput Biology Facility, University of British Columbia, Vancouver, BC Canada

**Keywords:** Lung adenocarcinoma, Bacteria, Microbiome, Methionine restriction

## Abstract

**Background:**

Recent studies have uncovered the near-ubiquitous presence of microbes in solid tumors of diverse origins. Previous literature has shown the impact of specific bacterial species on the progression of cancer. We propose that local microbial dysbiosis enables certain cancer phenotypes through provisioning of essential metabolites directly to tumor cells.

**Methods:**

16S rDNA sequencing of 75 patient lung samples revealed the lung tumor microbiome specifically enriched for bacteria capable of producing methionine. Wild-type (WT) and methionine auxotrophic (metA mutant) *E. coli* cells were used to condition cell culture media and the proliferation of lung adenocarcinoma (LUAD) cells were measured using SYTO60 staining. Further, colony forming assay, Annexin V Staining, BrdU, AlamarBlue, western blot, qPCR, LINE microarray and subcutaneous injection with methionine modulated feed were used to analyze cellular proliferation, cell-cycle, cell death, methylation potential, and xenograft formation under methionine restriction. Moreover, C^14^-labeled glucose was used to illustrate the interplay between tumor cells and bacteria.

**Results/Discussion:**

Our results show bacteria found locally within the tumor microenvironment are enriched for methionine synthetic pathways, while having reduced S-adenosylmethionine metabolizing pathways. As methionine is one of nine essential amino acids that mammals are unable to synthesize de novo, we investigated a potentially novel function for the microbiome, supplying essential nutrients, such as methionine, to cancer cells. We demonstrate that LUAD cells can utilize methionine generated by bacteria to rescue phenotypes that would otherwise be inhibited due to nutrient restriction. In addition to this, with WT and metA mutant *E. coli*, we saw a selective advantage for bacteria with an intact methionine synthetic pathway to survive under the conditions induced by LUAD cells. These results would suggest that there is a potential bi-directional cross-talk between the local microbiome and adjacent tumor cells. In this study, we focused on methionine as one of the critical molecules, but we also hypothesize that additional bacterial metabolites may also be utilized by LUAD. Indeed, our radiolabeling data suggest that other biomolecules are shared between cancer cells and bacteria. Thus, modulating the local microbiome may have an indirect effect on tumor development, progression, and metastasis.

**Supplementary Information:**

The online version contains supplementary material available at 10.1007/s13402-023-00832-7.

## Introduction

Lung cancer is a leading cause of cancer-related deaths worldwide [1]. Recent studies have demonstrated the importance of interactions between lung tumors and their microenvironment (TME), particularly in the case of immune cells, where the therapeutic implications have profound impacts on treatment [2–4]. The tumor microbiome, another important component of the TME, has only recently come into focus despite long recognition of the role of gut microbiota in regulating immune system health and disease [5–7]. The impact of the rich diversity of commensal bacterial on the biology of human anatomic niches, including the lung parenchyma is beginning to gain appreciation [8–11].

Historically, the role of bacteria with regards to cancer has generally been understood to increase the risk of cancer onset and progression. *Mycobacterium tuberculosis*, the causative agent of tuberculosis has been implicated in lung cancer through chronic inflammation [12]. However recent studies have begun to unveil the importance of the interplay between tumors and our bacterial symbiotes. For example, a tumor-associated microbiome has been detected across multiple cancers (33 types), and the identification of microbial DNA in tumors has potential for the development of a clinical marker for disease detection [13]. Further, recent cancer microbiome models have shown a tumor-specific bacterial colonization that is distinct from the surrounding non-malignant tissue [14, 15]. Moreover, changes in bacterial composition can be attributed to a tumor-specific niche, driven by both a tumor and immune cell response, and these changes can dynamically interact with the surrounding host cells with consequences for both healthy and diseased states [16–18]. While bacterial antigens are known stimulators of the human immune system and have the capacity to activate a local immune response [19], the direct impact of microbiome interactions on cancer cells through nutritional provisioning or detoxification remains underappreciated [20–23].

Although the lungs have long been considered to maintain a low commensal bacterial load in healthy individuals, recent studies have identified interactions between the lung microbiome and lung disease, including in cancer [24]. The majority of lung cancers, including lung adenocarcinomas (LUAD), can be attributed to the practice of smoking, which – in addition to altering lung cell DNA, RNA and protein – weakens the integrity of the lung epithelial barrier, increases lung susceptibility to infection and bacterial colonization, and disturbs the balance of healthy lung commensals [15, 25]. However, our understanding of the precise bacterial composition of the lung tumor-resident microbiome is limited, and the influence of resident bacteria on lung cancers is only beginning to be considered. Dysbiosis, or changes in the microbiome structure, have also been observed in several studies of lung cancer biopsies, while the presence of specific bacterial species in the gut has been associated with response to checkpoint blockade in lung cancer patients [26]. Thus, while progress is being made on identifying changes in microbiome structure in lung cancer patients, more comprehensive data sets are needed to determine the functionality of the core taxonomic diversity in the tumor microenvironment. This is particularly important in identifying metabolic interactions between lung tumor and microbial cells within the TME that could serve as potential therapeutic vulnerabilities.

In this study, we identified a tumor-specific microbiome within lung adenocarcinoma patient samples and explored putative metabolic interactions between lung tumor and microbial cells within the TME. Specifically, we sought to test the hypothesis that, under conditions of limited nutrients, bacterial cells can contribute metabolites locally to support tumor cell growth and other malignant phenotypes. We find that the tumor-specific microbiome has the capacity to increase production of L-methionine, an essential amino acid required for cancer cell growth, translation of proteins and epigenetic modification. Through these studies, we propose a dynamic interplay between tumor and bacterial cells that selects for bacterial populations capable of L-methionine production within the TME, which in turn supports pro-tumorigenic phenotypes.

## Patient information and methods

### Patient information

Tumor and matched adjacent non-malignant tissues were obtained from the BC Cancer Research Centre (BCCRC) after written informed consent from the patients and approval from the University of British Columbia-BCCA Research Ethics Board. At the time of resection, tissue samples were frozen in liquid nitrogen. Samples were subsequently stored in and retrieved from the Tumor Tissue Repository of the British Columbia Cancer Agency or Vancouver General Hospital. The disease margin was assessed by a pathologist after staining with hematoxalin and eosin. Tumor sections were then macrodissected to regions containing at least 70% tumor cell content. RNA and DNA was extracted from tumor and non-malignant (NM) slides as previously described [27]. Clinical information (% positive, Table 1) was calculated as a fraction of number of patients for which data was available in each clinical category. Gene expression and survival data (Illumina HiSeq) for The Cancer Genome Atlas cohort was downloaded from Cancer Browser (https://genome-cancer.ucsc.edu/proj/site/hgHeatmap/). Survival data was adjusted for clinical cofactors using a multivariate Cox Proportional Hazards model, and patient survival was not found to be associated with any clinical factor other than pathological stage (TCGA, *p* = 2.13 × 10^–8^). Gene expression and survival data for the KMplotter dataset is described in Nagy et al*.* [28].

**Table 1:** Clinical summary of discovery cohort

Cohort	BCCA		TCGA	
Clinical Feature	*N* = 77	%	*N* = 484	%
Age, mean (SD)	63.6(+ 9.8)		65.7(+ 9.7)	
Cancer Stage				
I	51	66.23	260	53.72
II	14	18.18	111	22.93
III	9	11.69	82	16.94
IV	3	3.90	24	4.96
Sex				
Male	24	31.17	263	54.34
Female	53	68.83	221	45.66
Smoking Status				
Current	32	41.56	47	22.23
Former	24	31.17	133	63.03
Never	21	27.27	51	14.69
*No data*			273	56.40
Mutation Status				
EGFR	13	16.88	33	14.35
KRAS	16	20.78	68	29.57
WT	11	14.29	130	56.52
*No data*	37	48.05	254	52.48

### Tissue profiling of human cells

DNA and RNA were extracted and processed as described above. After DNA and RNA extraction, tissue profiling was performed as previously described [27]. Briefly, regarding methylation profiling, DNA was bisulphite-converted and hybridized to the Illumina Methylation 27 K array (Illumina). Raw methylation values were corrected and normalized using SSN normalization using the Bioconductor lumi package in R [10]. Probe-specific methylation values were calculated by assessing the mean and standard deviation of each Beta value in the tumor and normal groups, and then assessing the difference in Beta-values of probes corresponding to LINE- and SINE-specific loci.

Regarding RNA analysis, all tumors from the BCCRC cohort were profiled for gene expression using the Illumina HT-12 Whole Genome 6 v3 BeadChip array (Illumina, San Diego, CA, USA). Raw data was corrected using the R mbcb package (version 2.11.0) [27], was then normalized and log transformed. Further, a subset of these (*n* = 36 pairs) were profiled by RNA sequencing on the Illumina HiSeq 2000 at the BC Cancer Genome Sciences Centre following library construction and barcoding [29].

### In-vivo tumor model

5 × 10^5^ luciferase expressing H2009 cell lines were subcutaneously injected into the right flank of 8-week-old, male, NRG mice. When tumors first became palpable, mice were separated into two groups: one fed either a methionine high diet (0.86% methionine) or a methionine restricted diet (0.11% methionine). Tumor size was measured by both digital calipers and luminescence for a total of 28 days. Animals were euthanized and tumors were resected.

### Cell line experiments

Human LUAD cell lines (A549, H2009, and H2030) used for in vitro and in vivo studies were obtained from American Type Culture Collection (ATCC, Manassas, VA, USA) and were cultured according to ATCC recommendations. Cells were maintained in RPMI-1640 (Gibco-ThermoFisher, Waltham, MA, USA), supplemented with 10% FBS, and grown at 37 degrees in a humidified 5% CO_2_ incubator. For methionine deprivation and titrated experiments, cells were switched to RPMI without methionine added (ThermoFisher Scientific, #A1451701) and supplemented with 10% FBS (ThermoFisher Scientific). Sterile L-methionine (99.0–101.0% pure, suitable for cell culture) was obtained and reconstituted in sterile distilled H_2_O to supplement the methionine-depleted media (Millipore Sigma, #M5308). 2-amino-bicyclo[2.2.1]heptane-2-carboxylic acid (BCH) was purchased through Cayman Chemicals (#15,249). LAT1 gRNA: UGUCUCCACAGUGCUGCUCA.

### Cell proliferation

Cell lines were seeded at a confluency of < 1000 cells/well in 6-well dish. Cells were incubated for 10 days, wherein media was changed on day 7. Cells were stained with Coomassie Brilliant Blue staining and washed using 1 × PBS. Colonies were analyzed using ImageJ plugin, ColonyArea [30]. Cell proliferation was also measured using AlamarBlue. Fluorescence was measured for 4 days, and data was normalized using fluorescent units recorded at Day 0.

### Cell cycle analysis

Cell lines were seeded in media with increasing concentration of methionine for 3 days. During the final 6 h, cells were incubated with BrdU at a final concentration of 10 µM. Cell staining was performed using BD Bioscience APC BrdU Flow kit (#552,598) and analyzed through flow cytometry.

### Histone extraction

Cell lines were grown in media containing either increasing concentration of methionine or filter-sterilized bacterially supplemented media. Cells were harvested and lysed with TEB Buffer (PBS containing 0.5% Triton X 100 (v/v), 2 mM phenylmethylsulfonyl fluoride (PMSF), 0.02% (w/v) NaN3). Nuclei was resuspended in 0.2N HCl for 16 h in 4 °C. Supernatant was neutralised using 2 M NaOH at 0.1 volume.

### Bacterial competition assay

To assess bacterial population abundance, all samples were thawed, washed with PBS, and stained with DAPI to distinguish cells from debris. Using an Attune NxT Acoustic Focusing Cytometer (Invitrogen), relative abundances of GFP and RFP positive cells in each sample were determined. Each measurement was assessed in biological and technical duplicate.

### Radiolabeling of cell lines

For determining whether a crosstalk exists between bacteria and cancer cells, we utilized C^14^ uniformly labeled glucose. Cells, either bacteria or LUAD, were grown in 0.1 µCi/mL RPMI-1640 media for 24 h for bacteria and 72 h for LUAD. Cells were collected and washed with PBS before being placed in unlabeled RPMI-1640 for an additional 24 h. The new labeled RPMI-1640 was centrifuged, followed by filter sterilization before being used. Cell lines were grown in newly labeled RPMI-1640 media before being collected, washed, and lysed with RPMI-1640 containing 0.1% SDS.

## Results

### Phylum-level shifts in microbiome between tumor and adjacent non-malignant samples

**Fig. 1:**
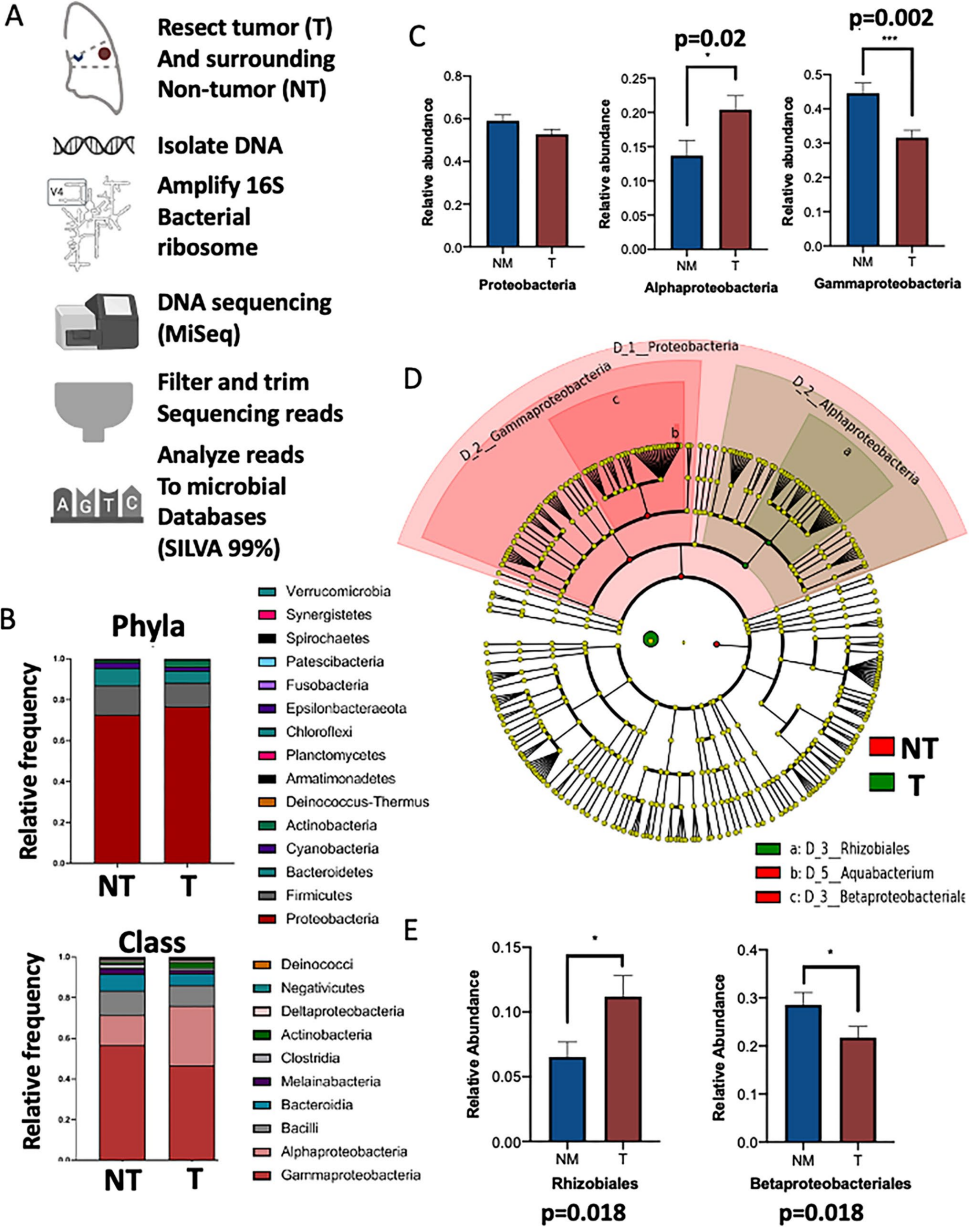
Lung adenocarcinoma tumors are enriched in alphaproteobacteria. **A**) Pipeline for analysis of 16S rRNA gene sequences recovered from human lung adenocarcinoma (tumor, red) and adjacent non-malignant (normal, blue) tissue. **B**) Taxonomical profiling of tumor- and NM microbiomes reveals that Proteobacteria are the most dominant bacterial phylum in both sample groups. Alphaproteobacteria and Gammaproteobacteria are the most abundant classes with reciprocal abundance between sample groups **C**) Relative abundance of Proteobacteria, Alphaproteobacteria, and Gammaproteobacteria in tumors and adjacent non-malignant tissues. **D**) Linear discriminate analysis Effect Size (LEfSe) between tumor and adjacent non-malignant samples (Kruskal-Wallis alpha value < 0.05, one-against-all analysis). **E**) Relative abundance of most significant classifiers between tumor and adjacent non-malignant tissues by LEFSe

To assess the lung adenocarcinoma (LUAD)-resident microbial community, we analyzed the 16S RNA component of the bacterial ribosomal (rRNA) gene in tumors from 77 patients, 75 of which had paired non-malignant (NM) lung tissue taken at the surgical margin (at least 3 cm away from the tumor margin) (Table 1, Fig. 1A). Samples were filtered for sequence quality (Supplemental Fig. 1A–B) and aligned to a phylogenetic tree composed of known 16S rRNA sequences using a de novo approach [31]. Extraction negatives (samples without lung tissue) were used as controls to exclude reads attributable to bacterial contamination from the DNA extraction process. Validation of bacterial species diversity and abundance was performed in a second, independent cohort from The Cancer Genome Atlas using LUAD tumor RNA sequencing reads depleted of sequences that align to the human genome [32].

Data generated above was used to evaluate microbial community diversity (alpha diversity) between non-malignant lung and tumor tissue from the same patient. According to four different alpha diversity metrics, which evaluate the species diversity within a microbial community, no significant change in alpha diversity was observed between tumor and surrounding NM tissue samples (Supplemental Fig. 1C). This observation aligns with previous studies in other cancer types that indicate changes in alpha diversity are only observed when tumor samples from cancer patients are compared to non-cancerous hospital control biopsies [32]. Additionally, Principal Coordinates Analysis of 16S rRNA Bray-Curtis distance did not show distinct clusters (i.e., no clear separation) between tumor and adjacent NM samples (PERMANOVA *p*-value = n.s.), indicating that interpatient similarity may be stronger than similarity between tumors (Supplemental Fig. 1D). While the degree of microbiome diversity does not differ significantly between tumor and NM lung tissue, shifts in microbiome structure is evident regarding relative abundance of bacterial populations.

Based on these results, we hypothesized that specific subsets of microorganisms would preferentially colonize the tumor or NM tissues due to niche differences, including potentially localized metabolic dependencies of the TME. As with the microbial profiles of other solid cancer types, we observed a dominance of *Proteobacteria* and *Firmicutes* at the phylum level in both the LUAD tumor and NM samples (Fig. 1B). At the class level, the bacterial community structure of both cohorts was primarily comprised of *Alphaproteobacteria* and *Gammaproteobacteria *(Fig. 1B). Importantly, we observed a significant inversion in the relative abundance of these taxa between tumors and their matched normal samples, as the tumor group was predominantly enriched for *Alphaproteobacteria* (*p* = 0.0028), whereas the NM group showed a significantly higher relative abundance of *Gammaproteobacteria* (*p* = 0.0007) (Fig. 1C). Additionally, we did not observe any detectable reads classified as *Streptococcus pneumoniae* in these patients, which accounts for the majority of community-acquired pneumonia cases, suggesting that the observed bacterial abundances were not driven by acute infection [33] (Supplemental Table 1).

To determine if observed phylum- or class-level bacterial community shifts are sufficient to distinguish LUAD tumor from adjacent NM sample groups, we employed a linear discriminant analysis effect size (LEfSe) model [34]. Briefly, LEfSe is a statistical model used for biomarker discovery that combines a Kruskal-Wallis non-parametric sum-rank test with a linear discriminant analysis to identify genomic features important to classification between biologically distinct groups. Using this method, we observed significant shifts in *Proteobacteria* phyla differentiating tumor from adjacent NM tissues (Fig. 1D, Supplemental Fig. 2A). Specifically, increases in the relative abundance of the *Alphaproteobacteria* class defined tumor samples while increases in *Gammaproteobacteria* were predominant in NM tissues (Fig. 1D, Supplemental Fig. 2A). Of note, the most predictive taxa were *Alphaproteobacteria* (*Rhizobiales* order) and *Deinococcus* in tumors, and *Gammaproteobacteria* (*Betaproteobacteriales* order) in NM samples (Fig. 1D–E, Supplemental Table 1). These observations were independent of tumor stage in the BCCRC cohort (*Alphaproteobacteria* and *Gammaproteobacteria* classes shown, Supplemental Fig. 2B).

### Enrichment of microbial biosynthetic pathways implicate methionine as a lung tumor specific microbial derived metabolite

While shifts in the relative abundance of specific microbial taxa can impact host cell metabolism, horizontal gene transfer and functional redundancy can offset these changes at a community level [35, 36]. Thus, knowledge of gene function is critical to reconstructing metabolic networks as metabolic changes in the microbiome can affect the host by regulating the availability of key substrates for host cell metabolic processes [16]. In the absence of whole genome sequencing (shotgun metagenomics) information and in tissues with low bacterial loads, 16S rRNA gene sequences can be used to infer unobserved metabolic states when mapped to cognate reference genomes [37]. We used PICRUSt2 to predict metabolite profiles produced from the tumor and NM tissue microbiomes, which uses 16S sequencing data to assign metabolic capabilities of unannotated genomes to their nearest annotated neighbour in phylogenetic space [37].

We then assessed the metabolic differences between the presumed bacterial metagenomes in tumors compared to those in the surrounding NM tissues and then assessed enrichment of the identified compounds in KEGG and MetaCyc pathways to evaluate their potential impact on host biology. We identified 13 pathways with significantly different prominence between tumor and NM tissue microbiomes (|FC|> 1.3, B-H *p* < 0.05; Table 2, Supplemental Table 2). Seven of these pathways appeared to be more active in tumor microbiomes, while six were predicted to be less active. We found that these metabolic functions were enriched in pathways producing L-methionine (Table 2); specifically, the tumor-associated microbiome appeared to overproduce L-methionine through glycine betaine degradation and decrease bacterial use of S-adenosyl-L-methionine (SAM) (Supplemental Fig. 3A–B). Interestingly, we found that *Rhizobiales*, and not *Deinococcus,* were among the top 5 bacterial strains responsible for the methionine enrichment. Together, these results suggest that the tumor microbiome overproduces L-methionine relative to NM tissue microbiome, which we hypothesized could promote tumor progression, especially under conditions when nutrients within the TME become limited.

**Table 2:** Tumor microbiome predicted to upregulate pathways involved in epigenetic processes

Direction (tumours)	Pathway #	Fold Change	BH *p*-value*
Up	Glycine Betaine degradation ^M1^	46.4	0.002
	Nylon-6 oligomer degradation ^M2^	2.1	0.001
	Bifidobacterium shunt ^A3^	1.9	< 0.001
	Ketogluconate metabolism ^4^	1.8	< 0.001
	Pyruvate fermentation to aceton ^A5^	1.7	< 0.001
	Acetyl-coA fermatiation to butanoate II ^A6^	1.6	0.003
	Photorespiration ^7^	1.5	0.003
Down	S-adenosyl-L-methionine cycle I ^M−6^	0.7	0.002
	2-methylcitrate cycle I ^A−5^	0.68	0.002
	L-Glutamate and L-glutamine biosynthesis ^−4^	0.65	< 0.001
	NAD biosynthesis II^−3^	0.63	0.002
	Pyridoxal 5'-phospahe biosynthesis I ^M−2^	0.6	0.001
	GDP-D-glycero-alpha-D-manno-heptose biosynthesis ^−1^	0.1	0.002

^*^All pathways differentially predicted to be involved in the metabolism of the tumour microbiome ($\left|\mathrm{FC}\right|$>1.3, Benjamini–Hochberg corrected *p*-value < 0.05). # Pathways associated with epigenetic modifiers are labelled as: methylation (M) or acetyl group donation (A)

### Cancer cells generate selective pressure for bacteria that produce L-methionine in a model microenvironment system

**Fig. 2:**
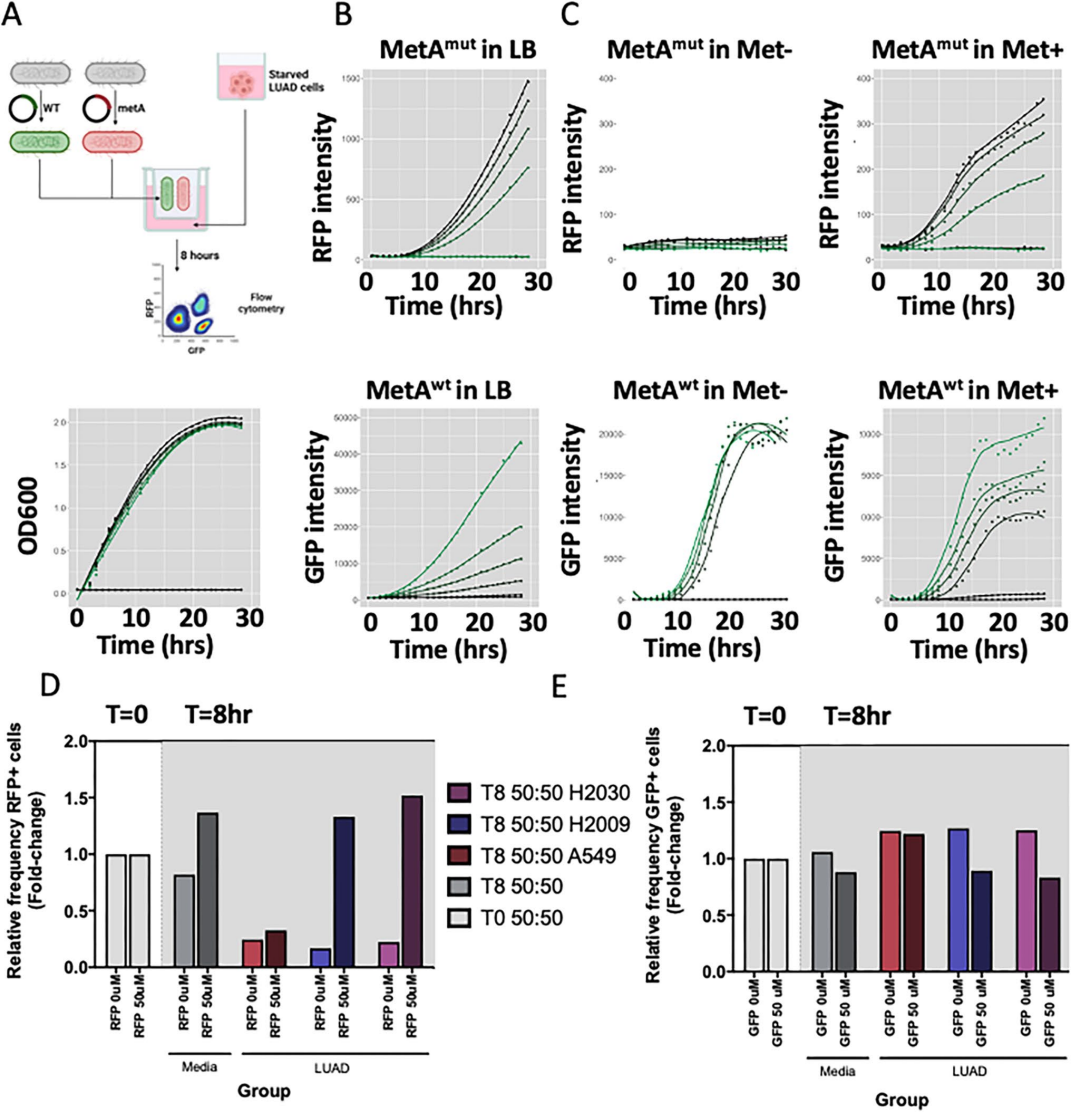
Co-culture of methionine-producing and non-producing *E. coli* strains in the context of lung adenocarcinoma (LUAD) cells reveals ability of LUAD cells to select for growth. **A**) Generation of parental wild-type (metA WT) *E. coli* (GFP-expressing) and auxotrophic metA mut *E. coli* (RFP-expressing) strains derived from the Keio collection of K-12 auxotrophs (Left). Growth of co-cultures in with LUAD and varying ratios of inoculation (Right). **B**) metA mut (RFP) and metA WT (GFP) strain growth in LB over time at varying inoculation fractions. **C**) Growth of metA mut and metA WT strains at varying inoculation ratios in M9 minimal media without the addition of L-methionine (Met-) (0 mM, white panel), or with L-methionine supplementation (Met+) (50 mM, grey panel). **D**) Relative fraction of RFP + *E. coli* cells and **E**) GFP + cells after 8 h of growth in LUAD growth media without (grey) and with exposure to LUAD cells (A549, H2009, and H2030). Culture without methionine supplementation is shown as light bars, while cultures with methionine supplementation are shown as darker bars

Given that the taxonomic data revealed a preponderance of methionine-producing bacteria in the lung cancer microbiome, we next sought to investigate if the tumor niche could select for enrichment of methionine-producing bacteria. We hypothesized that nutrient deprivation in the lung TME could act as a selective pressure for methionine-producing bacterial strains, consequently supplying methionine to tumor cells. To address this question, we performed bacterial competition assays using co-culture experiments with cancer cells to assess the fitness of *E. coli* strains with different abilities to produce methionine. Firstly, we established a fluorescent tracking system for the bacterial strains. A parental strain (*E. coli* metA^WT^, methionine producer) and methionine auxotrophic line (*E. coli* metA^mut^, non-producer of methionine from the Keio collections [38]) were transformed with GFP and RFP plasmids to generate *E. coli* metA^WT^-GFP and metA^mut^-RFP, respectively. The growth of each population over time at varying inoculation ratios can be determined by assessing relative fluorescence of the strains (Fig. 2A) [39]. In methionine-containing LB media, both the auxotrophic metA^mut^ and the metA^WT^ strain grew when inoculated at equivalent ratios, with the GFP/RFP ratio slightly favouring the auxotroph over time (Fig. 2B). When grown in minimal media (M9) supplemented with methionine, the fitness of the metA^mut^ strain was dependant on the concentration of methionine in the media for under all tested conditions (Fig. 2C, Supplemental Fig. 4). We determined that 50 µM methionine (equivalent to the concentration in the common LUAD growth media RPMI-1640) allowed for growth of both bacterial populations (Fig. 2C, Supplemental Fig. 4).

Due to the highly proliferative nature of lung tumor cells, we anticipated that the TME would become depleted of methionine. We utilized the above-mentioned LUAD cell lines with the *E. coli* metA^WT^-GFP and metA^mut^-RFP co-culture systems to model the competitive environment for methionine availability (Fig. 2A). After LUAD cell starvation, bacterial cultures (at a 50:50 ratio) were added above a sterile transwell in the presence (50 µM) or absence of methionine. After growth, co-cultures were sampled, and the relative proportion of each bacterial population was measured by flow cytometry (Supplemental Fig. 5A–B). Over time, we observed that the bacterial population shifted towards the metA^WT^ strain faster in the presence of LUAD cells than in cell culture media without LUAD cells (Fig. 2D). When methionine was present at 50 µM, this shift did not occur (Fig. 2E). These results indicate that LUAD cells played a direct role in generating a methionine-deprived microenvironment that favours methionine producers over non-producers, as in our model system, microbes that produced their own methionine were able to out-compete those unable to produce methionine.

### Bacterial-produced methionine restores LUAD phenotypes under low nutrient stress

**Fig. 3:**
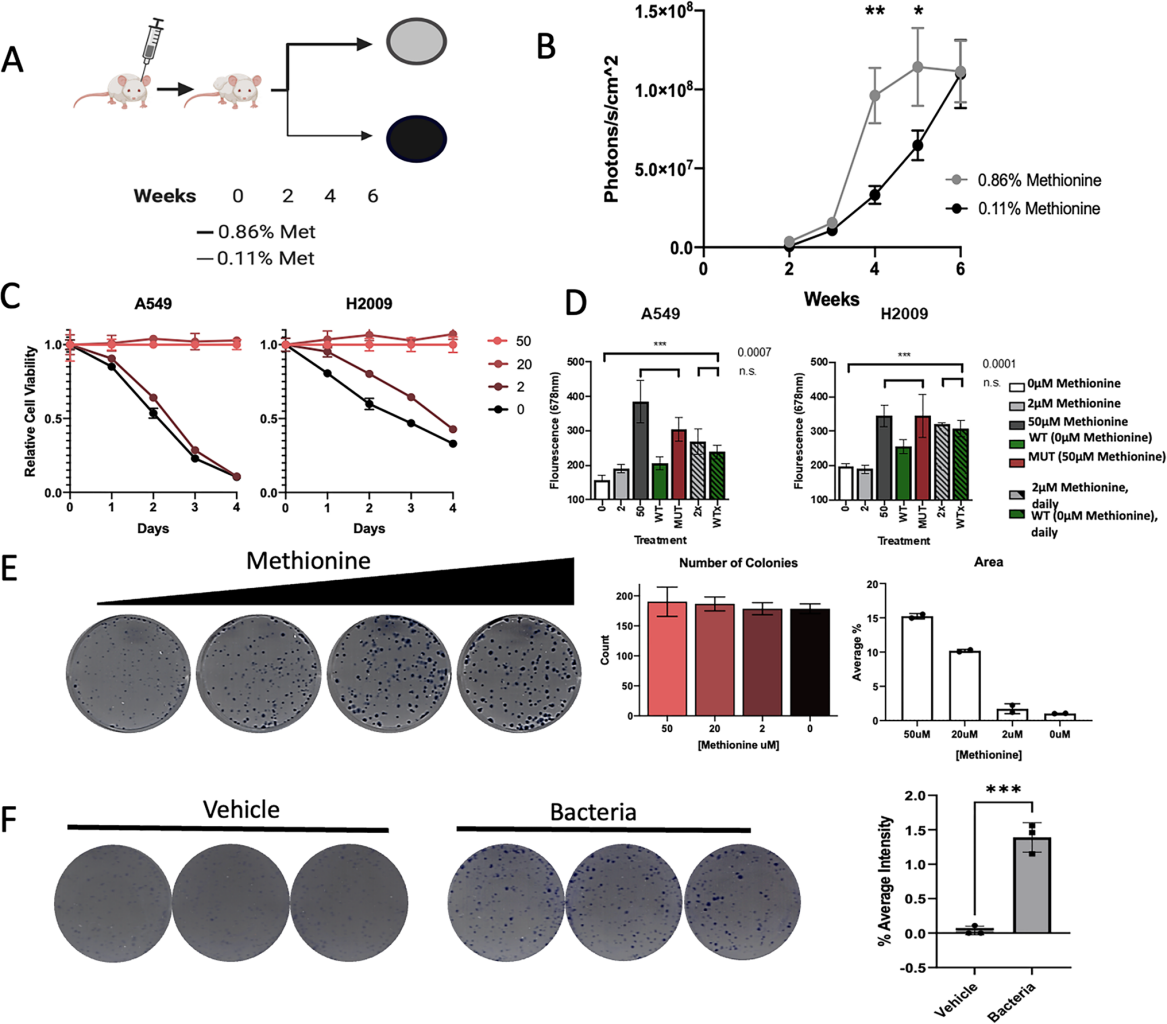
Bacterial Synthesized Methionine can Rescue Cancer Phenotypes. **A**) Schematic for in-vivo methionine restriction study. Mice were sub-cutaneously injected with luciferase expression LUAD cell lines and placed on either methionine high (0.86% methionine) or methionine low (0.11% methionine) diets. **B**) Tumor growth of in vivo experiment with animals in methionine high and methionine low diet. Photons were collected using AMI Imager after intraperitoneal injection of luciferase into animal. **C**) AlamarBlue analysis measuring relative fluorescence units across multiple days and decreasing levels of methionine in the media. **D**) Proliferation assay of cancer cells in decreased levels of methionine and the presence of bacteria, using WT or metA MUT E. coli cells. Hashed bars denote LUAD cell lines grown with daily changes of respective media. **E**) Clonogenic assay and data analysis of cancer cells under decreasing levels of methionine (0, 2, 20, and 50 µM methionine). Quantity of colonies formed was counted manually due to low levels of methionine resulting in faint staining. Area of colonies was determined by staining intensity measured by ImageJ. **F**) Clonogenic assay of cells under low methionine media in the presence or absence of bacteria. Area of colonies was determined by staining intensity measured by ImageJ

Bacteria have a dedicated pathway responsible for synthesizing methionine de novo, whereas mammalian cells lack this pathway and must rely on an outside source for this nutrient, typically acquired from their diet*.* With the proximity of the microbiome within tumors, and the tumors selective pressure to support high methionine producers, it is possible that bacteria could act as a secondary source for necessary nutrients. To understand and study how methionine producing bacteria may be able to alter LUAD growth and other malignant phenotypes, we first aimed to determine how LUAD cells would respond in vivo and in vitro to limited methionine levels. We performed an *in-vivo* experiment using NRGS mice that were subcutaneously injected with LUAD cells and placed on methionine high (0.86% methionine) or low (0.11% methionine) diets (Fig. 3A). We tracked tumor growth weekly using bioluminescence and observed a reduction in growth rate for mice placed on the methionine low diet (Fig. 3B).

Our findings were recapitulated *in-vitro* through cell proliferation assays where methionine concentrations of 2 µM and 0 µM yielded drastic reduction in proliferation of LUAD cells compared to those grown at higher concentrations (Fig. 3C, Supplemental Fig. 6A). The reduction in proliferation was not due to an induction of apoptosis, as we recorded no significant difference in Annexin VI staining (Supplemental Fig. 6B). As such, we investigated whether the decreased proliferation and non-significant changes in apoptosis observed was due to arrest in the cell cycle. Indeed, our BrdU results show a G2 cell-cycle arrest for cells under methionine restriction (Supplemental Fig. 6C). In addition to proliferation, we measured motility by wound healing assay which demonstrated that lowering methionine concentrations lessens cell migration (Supplemental Fig. 6D); however, these findings were not validated in live imaging assays (Supplemental Fig. 6E). We hypothesized the decrease in wound healing capability was a result of decreased proliferation and not of decreased motility. By investigating the wound healing property under live-cell microscope, it is clear that methionine restriction’s impact on proliferation is having a direct impact on wound healing (Supplemental Video 1).

As we observed an elevation of methionine synthesis, we investigated whether bacterially produced methionine could be used as a supplement in nutrient poor TME environments. At lower concentrations of methionine, the metA^mut^ strain was unable to rescue the proliferation of LUAD cell lines (Fig. 3D; Unpaired t-test, *p* = 0.0007, 0.0001, and 0.0017), presumably due to a lack of available methionine, while the WT strain was capable of rescuing proliferation of LUAD cell lines (Fig. 3D). This was further supported by clonogenic assays. We seeded equal number of LUAD cells and allowed them to grow over 10 days and saw a decrease of colony size, as denote by the intensity of the stain (Fig. 3E). Interestingly, bacterially supplemented media (Fig. 3F) or returning methionine concentration in the media to adequate levels (Supplemental Fig. 7A–B) demonstrated a rescued proliferation phenotype. Taken all together, these data would suggest LUAD cell lines are able to utilize the methionine synthesized by the bacteria.

### Patients with tumors highly expressing essential amino acid transporter LAT1 have poorer overall survival

**Fig. 4:**
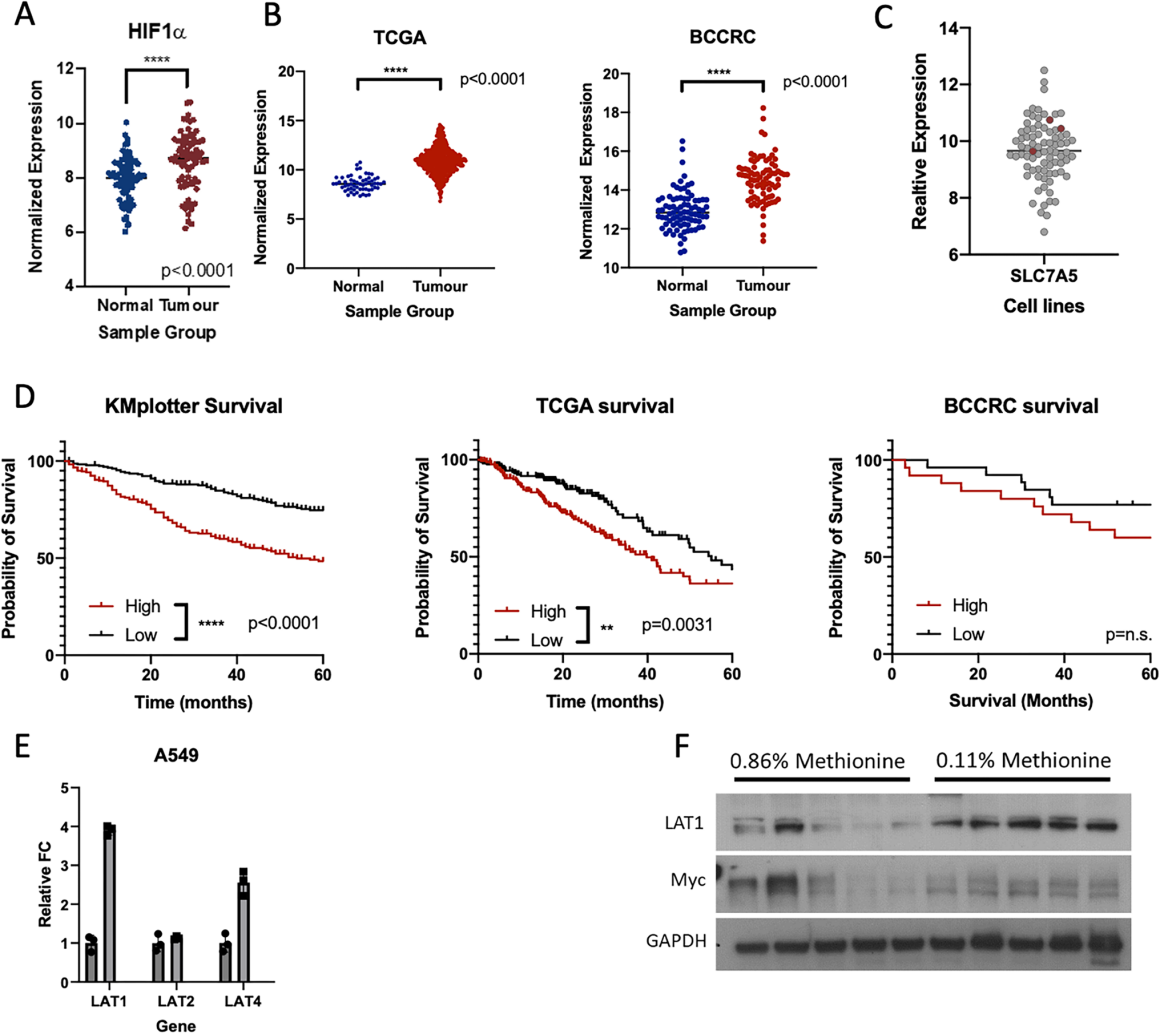
Nutrient stress elevates LAT1 transporter expression. **A**) Relative expression of HIF1alpha in tumor and NM samples (paired t-test, *p* < 0.0001). **B**) Expression of LAT1 in patient samples. **C**) Expression of LAT1 across multiple LUAD cell lines. **D**) Kaplan Meier plot of survival in patients with lung cancer and high vs low expression of LAT1 mRNA. **E**) qPCR analysis of A549 and H2009 under methionine high (50 µM) and methionine low (2 µM) after 48 h incubation. **F**) Western blot analysis of in-vivo tumor experiment (Fig. 3a) probing for anti-LAT1 and anti-Myc antibody.

In response to an environment with limiting nutrients, specific transporters are upregulated to restore adequate levels of nutrients intracellularly. To assess if the TME is nutrient-deprived, we assessed the relative expression of the *HIF1a* gene, an established marker of hypoxic signaling. We found higher expression of HIF1a in tumors compared to their matched NM samples, confirming expected tumor hypoxia, and suggesting potential nutrient deprivation (Fig. 4A). Our earlier findings suggest bacterially supplemented methionine sufficient for partial rescue of proliferation, leading us to investigate the expression levels of a known methionine transporter, LAT1 (SLC7A5) in tumor and NM tissue samples. Using the TCGA and BCCA cohorts and found that tumors overexpressed *SLC7A5* in both cohorts (*p* < 0.0001, unpaired [A] and paired [B] T-test, respectively) (Fig. 4B). Moreover, LUAD cell lines exhibit high LAT1 expression (Fig. 4C). To determine if upregulation of this transporter was important to patient outcome in LUAD, we assessed the levels of *SLC7A5* in three LUAD datasets; BCCRC (*n* = 77 pairs), TCGA RNA-seq (*n* = 562; 503 tumor, 59 non-malignant), and KMplotter (*n* = 720 tumor) (Table 1). When separated into tertiles according to *SLC7A5* tumor expression, we found that patients with elevated *SLC7A5* had worse overall survival than patients with low levels, supporting a relationship between neutral amino acid uptake—including methionine—and tumor progression[40] (Fig. 4D). This relationship between LAT1 expression and methionine restriction was further validated in our qPCR data, showing increased levels of LAT isoforms 1 and 4, but not LAT2, in LUAD cell lines (Fig. 4E). Furthermore, mice placed on a low methionine diet had elevated levels of LAT1 expression compared to those on a high methionine diet (Fig. 4F). While attempts were made to KO LAT1 using CRISPR guide RNA (Supplementary Fig. 8A), LUAD cell lines were unable to be reseed following trypsinization. We then used 2-amino-bicyclo[2.2.1]heptane-2-carboxylic acid (BCH), a known LAT1 inhibitor. We saw no change in cellular proliferation by alamarBlue using 100 µM BCH treatment, a concentration reported to affect amino acid uptake [14] that did not impact survival, in either 50 µM or 2 µM of methionine (Supplementary Fig. 8B and C). These data suggest that the TME is limited in nutrient availability and that expression of specific LAT transporters, including LAT1, are elevated under nutrient stress.

### Biomolecules are exchanged between cancer cells and microbiome

With the changes observed in our *in-vitro* data investigating how bacterially supplemented media can impact proliferation, we next sought to identify how bacteria may impact signaling. Mammalian cells react to nutrient availability through stimulation of cell signaling, most notable through the mTOR pathway. Similar to previous literature, we observed increased phosphorylation of AKT under methionine deprivation [41] (Supplemental Fig. 9A–B). Interestingly, supplementing LUAD cell lines with bacterially grown media resulted in a decrease in phosphorylation of AKT and an increase in phosphorylation of ERK signaling (Supplemental Fig. 9A–C). These results are surprising as both AKT and ERK phosphorylation are understood to increase cellular proliferation. Clearly, there is a possible crosstalk between both organisms, wherein the tumor cells select for specific bacterial families and the bacteria, in turn, supply the tumor with nutrients and impact signaling.

**Fig. 5:**
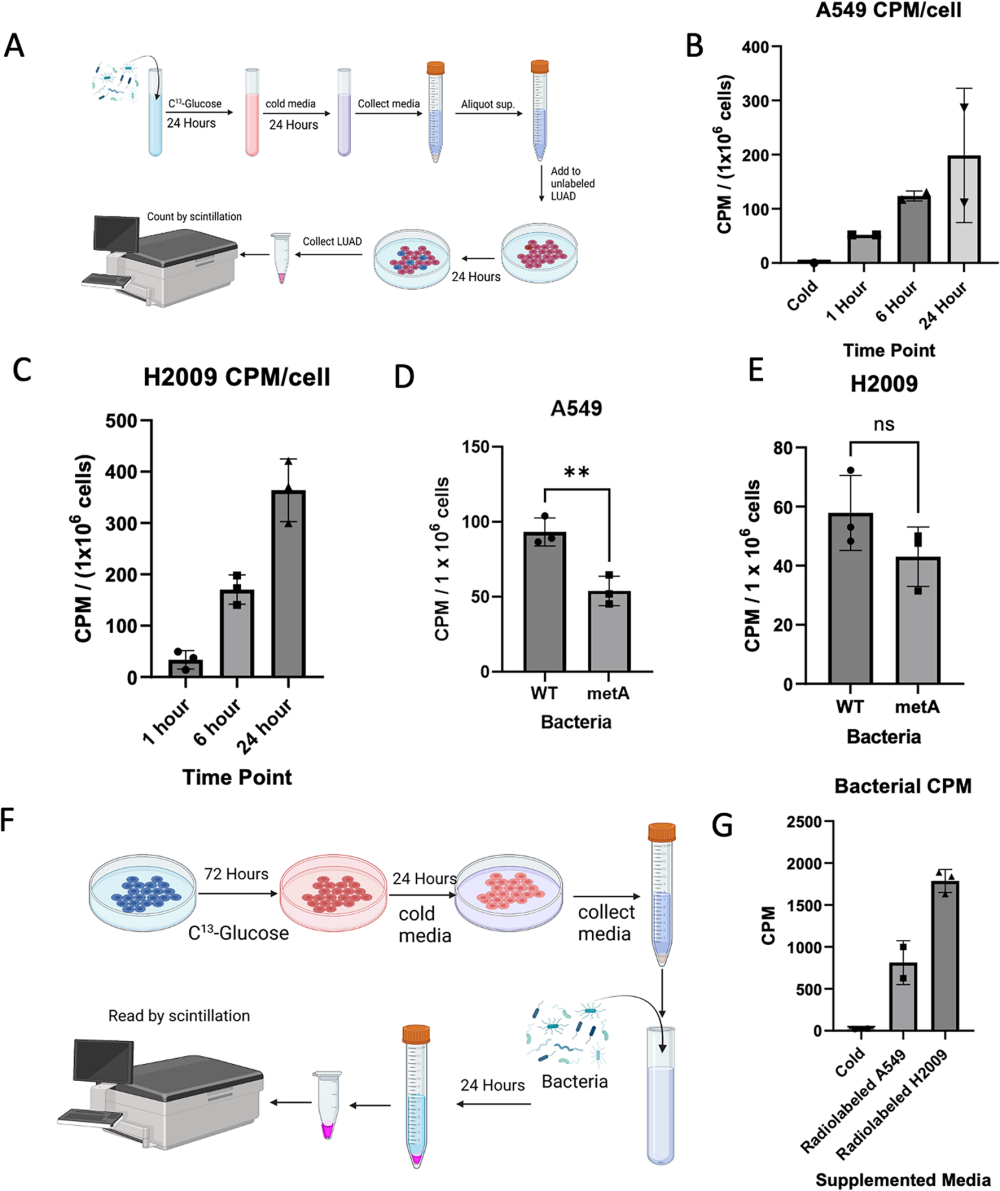
Crosstalk between LUAD and the microbiome. **A**) Schematic for radiolabeling LUAD cell lines with bacterial metabolites. Uniformly labeled C-13 Glucose was used to label bacterial cells. **B**) Scintillation counter CPM for A549 incubated in either no bacterially labeled supplemented media (Cold) or bacterially labeled supplemented media after 1, 6, and 24 h. Data was normalized based on cell count. **C**) Scintillation counter CPM for H2009 incubated with bacterially labeled supplemented media for 1, 6, and 24 h. Data was normalized based on cell count. **D**) Scintillation counter CPM for A549 and @) H2009 WT vs metA mutant supplemented media. Following overnight incubation of cold media, WT E. coli was diluted with cold media until OD600 matched metA. **F**) Schematic for radiolabeling bacteria with LUAD metabolites. Uniformly labeled C-13 Glucose was used to label LUAD cell lines. **G**) Scintillation counter CPM for bacteria following 24 h of incubation in LUAD supplemented media, with cold being LUAD unlabeled supplemented media

To further investigate the crosstalk between LUAD and the microbiome, we performed radiolabeling experiments. WT *E. coli* were uniformly labeled with C^14^-glucose and placed in unlabeled RPMI-1640 media to generate our “Bacterially labeled RPMI-1640 media” (Fig. 5A). LUAD cells treated with the newly bacterially supplemented media became radiolabeled in a time-dependent manner (Fig. 5B–C). As our data relied on methionine as a tool for nutrient restriction, we next tested whether metA mutant *E. coli* could label LUAD cells under conditions of methionine restriction. While metA mutant *E. coli* supplemented media labeled LUAD cells under methionine restriction, the labeling observed was significantly less than that of WT *E.* coli (Fig. 5D–E). We next sought to identify whether this interaction was unidirectional or bidirectional. Interestingly, we found that radiolabeled LUAD supplemented media (Fig. 5F), was also capable of labeling bacteria cells (Fig. 5G). All together, these results would indicate there exist a dialogue between cancer cells and the surrounding microbiome wherein metabolites are shared between both organisms.

## Discussion

Recent studies on large-scale detection of microbes in human tumors have highlighted the understudied nature of the tumor-resident microbiome, indicating the need for a multidimensional approach to understand the interactions within these complex tumor-microbe systems [13, 42, 43]. These studies widen the window on biomarker discovery and the development of novel therapeutic applications based on targeting relevant microbiome functions. Here, using clinical specimens, we have demonstrated that, compared to NM tissue, the lung tumor microbiome has the potential to overproduce methionine with a concomitant reduction in SAM catabolism. This overproduction phenotype could provide a selective advantage in the TME niche-space where methionine is limited due to rapid tumor cell proliferation or decreased nutrient delivery caused by defective vasculature. Moreover, methionine producing populations can, in turn, provision tumor cells, creating a positive feedback loop in tumor progression. This phenotype is not restricted to a specific strain and can likely be transferred across multiple taxa under selection in the TME. Beyond characterizing the importance of specific bacterial species, this study supports the notion of an active and dynamic interplay between the resident bacterial community and the lung TME, reinforcing the importance of considering the influence of the microbial community, rather than limiting our observations to variations in specific bacterial species.

While nutrient restriction, in general, has been shown to affect cancer cell growth and proliferation, we sought to understand cellular adaptation in nutrient restriction in more detail. We observed upregulation of methionine transporters in tumor cells, alteration in epigenetic status, and increased methionine production by the LUAD microbiome, highlighting the importance of this essential amino acid to lung tumor biology. Indeed, we observed that increasing the concentration of available methionine after starvation resulted in a release from cancer-cell-specific cell cycle blockade and allows for subsequent proliferation. The importance of methionine to cancer proliferation and growth has been found in other cancer types. In glioblastoma, methionine restriction has been shown to be limiting, and supplementation with methionine is able to dictate translation rate [44]. Our results indicate that the local tumor microbiome is a potentially undiscovered source of methionine that can directly impact tumor progression.

Upon observing an increase in methionine production by the tumor-resident bacterial community, we chose to model the effects of this pathway using engineered *E. coli* – a well-described and tactile member of the *Proteobacteria* phylum, which was the most abundant phyla in both tumor and adjacent NM samples. This allowed us to best characterize the effects of the methionine biosynthetic pathway on the tumor. We observed that transplanted tumor and LUAD cell line growth could be influenced by availability of methionine, and cell line proliferation could be rescued by methionine produced by *E. coli*. Further, using an in vitro bacterial co-culture system, we observed a shift in the bacterial population towards the wild-type (metA^WT^) methionine-producing strain relative to a mutant strain deficient in methionine production. The observed selection in cell culture systems indicates the possibility of a pressure on the bacterial community within human tumors, and that this selection in turn dynamically modifies the tumor microenvironment and contributes to cancer cell metabolism. Hence, these data raise the intriguing possibility that the tumor niche may be able to select bacterial species associated with a higher production of methionine.

There have been very few studies of microbial lung tumor microbiomes, and these have primarily focussed on describing bacterial diversity in cancer subtypes [15, 32, 45]. Our data revealed higher relative abundance of *Deinococcus* in tumor samples compared to NM tissues, which is in keeping with previous observations in the squamous cell subtype of lung cancers (LUSC) (Supplemental Table 1) [32]. Our study presents a comprehensive view of the bacterial community in human lung cancer and supports the notion that microbiome population shifts can metabolically contribute to tumor cell growth. The metabolic contribution of bacteria was further supported by our radiolabeling studies, where bacterially supplemented media was sufficient to label LUAD cells. Our radiolabeling studies also suggested the interaction between the bacteria and LUAD cells exhibited a crosstalk between the two organisms. However, our study is not designed to differentiate between bacteria occupying the TME and those possibly internalized by tumor cells [23]. The fact that mammalian cells are incapable of synthesizing the essential amino acid methionine suggests that methionine dependency is a pan-cancer phenomenon, even though the tumor-resident microbiome is likely to vary depending on the condition of the tumor niche across cancer types. Additionally, labeling of the LUAD cell lines by bacterially supplemented media occurred regardless of nutrient restriction, suggesting other microbial biomolecules are present during the exchange. Whether these molecules are signaling molecules, growth factors, or other nutrients is yet to be determined.

In conclusion, we have presented data to support a dynamic interaction between tumor and tumor-resident bacterial cells. Specifically, our data supports that the LUAD TME exerts a tumor-specific microbial selection pressure on microbial communities that can in turn contribute to tumor metabolism through the production of methionine at the tumor site. Further, an association between the expression of a methionine transporter with patient survival, suggests therapeutic potential directed towards the bacterial component of the TME. Moreover, our findings indicate a possible crosstalk between the tumor and the surrounding microbiome that may rescue tumor survival under nutrient-deprived states. As nutrient-deprived phenotypes are common among solid tumors and the metabolic potential of the microbiome is broad, this work has wide-reaching implications for tumor behaviour across tissue types including a wide variety of tumor niches.

### Supplementary Information

Below is the link to the electronic supplementary material.

Supplementary file1 (XLSX 1252 KB)

Supplementary file2 (XLSX 540 KB)

Supplementary file3 (XLSX 10 KB)

Supplementary file4 (PDF 1421 KB)

Supplementary file5 (MP4 4.84 MB)

## Data Availability

The datasets generated during and/or analysed during the current study are available from the corresponding author on reasonable request.

## Abbreviations

NSCLC: Non-small cell lung cancer
LUAD: Lung adenocarcinoma
NM: Non-malignant
LUSC: Lung squamous cell carcinoma
OTU: Operational taxonomic unit
ASV: Amplicon sequence variant
