# Supplementary material for: Methionine-producing tumor micro(be) environment fuels growth of solid tumors
Source: Cell Oncol (Dordr). 2023 Jun 15;46(6):1659–73. doi: 10.1007/s13402-023-00832-7 (PMC10697899; doi:10.1007/s13402-023-00832-7)
Supplement: Supplementary file 4 — Supplementary file4 (PDF 1421 KB) [file 13402_2023_832_MOESM4_ESM.pdf]

Supplemental Information for:

Methionine-producing Tumor Micro(be) Environment fuels Growth of Solid Tumors

Alexis A. Vega[1,2,^], Erin A. Marshall[3,4,^], , Avery J.C. Noonan[5,6], Fernando Sergio Leitao Filho[7], Julia Yang[7], Greg L. Stewart[3,4], Fraser D. Johnson[3,4], Emily A. Vucic[8], Michelle E. Pewarchuk[3,4], Parag P. Shah[2], Brian F. Clem[1,2], Corey Nislow[9], Stephen Lam[3], William W. Lockwood[3,4,10], Steven J. Hallam[5,6,11-13], Janice M. Leung[7], Levi J. Beverly[2,#*], Wan L. Lam[3,4,10,#]

[1]Department of Biochemistry and Molecular Genetics, University of Louisville, Louisville, KY, USA
[2]Brown Cancer Center, University of Louisville School of Medicine, Louisville, KY, USA
[3]Integrative Oncology, BC Cancer Research Centre, Vancouver, BC, Canada
[4]Interdisciplinary Oncology Program, University of British Columbia, Vancouver, BC, Canada
[5]Genome Science and Technology Program, University of British Columbia, Vancouver, BC, Canada
[6]ECOSCOPE Training Program, University of British Columbia, Vancouver, BC, Canada
[7]Centre for Heart Lung Innovation, St Paul's Hospital, Vancouver, BC, Canada
[8]NYU Langone Medical Center, New York, NY, USA
[9]Faculty of Pharmaceutical Sciences, University of British Columbia, Vancouver, BC, Canada
[10]Department of Pathology and Laboratory Medicine, University of British Columbia, Vancouver, BC, Canada
[11]Department of Microbiology & Immunology, University of British Columbia, Vancouver, BC, Canada
[12]Bioinformatics Program, University of British Columbia, Vancouver, BC, Canada
[13]Biofactorial High-throughput Biology Facility, University of British Columbia, Vancouver, BC, Canada

*Corresponding author
[#] Senior author
^ Contributed equally

Correspondence:

Levi Beverly
Levi.Beverly@Louisville.edu
University of Louisville
Brown Cancer Center
505 S. Hancock St. rm 204
Louisville, KY 40202

Supplemental Figure 1

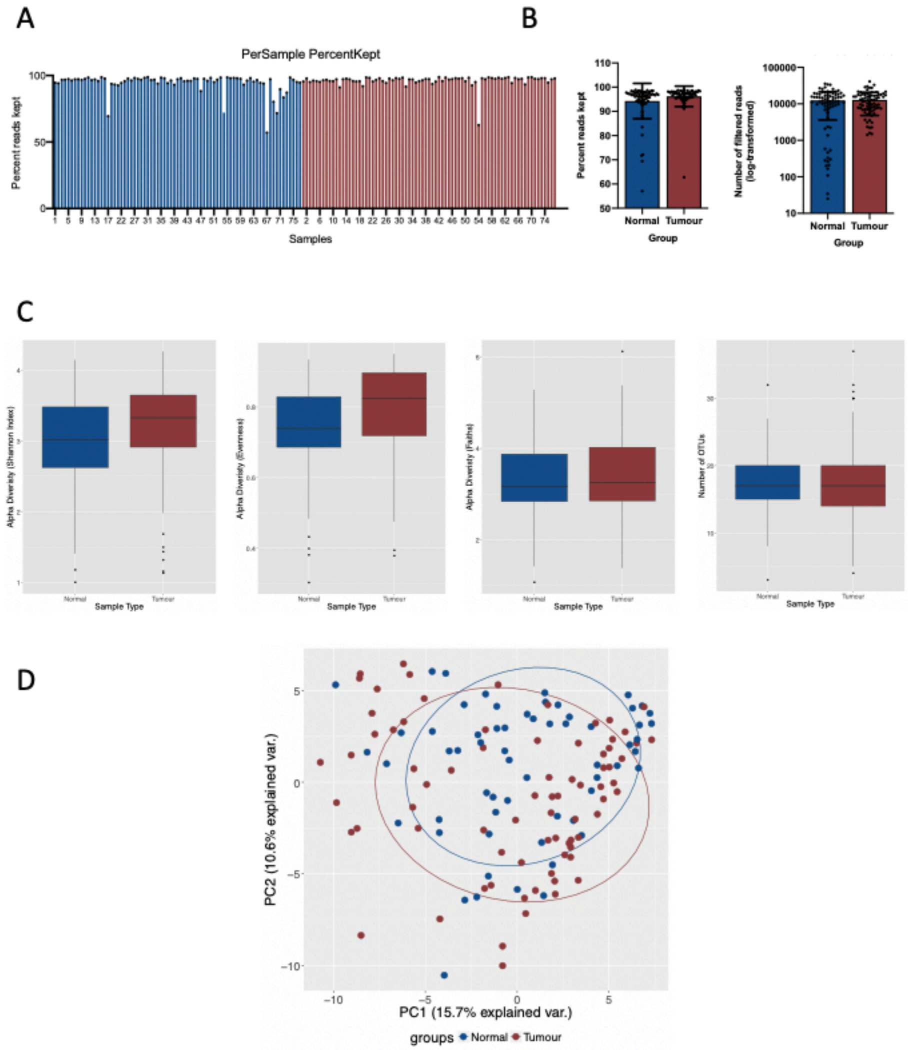

Supplemental Figure 1. Diversity of microbial communities in lung adenocarcinoma tumors and non-malignant regions. A) Percent of reads for downstream analysis after filtering sequences for read quality per sample. B) The fraction of reads retained after filtering and absolute number of reads remaining is not statistically different between the non-malignant and tumor samples. C) Alpha diversity of samples is not significantly different between non-malignant and tumor samples by 4 metrics: Shannon Index, Evenness, Faith PD (phylogenetic), and number of OTUs. D) Beta diversity analysis based on Bray-Curtis distance reveals that the microbial communities of non-malignant and tumor samples were not significantly different (i.e., no clear separation between samples of both groups). In box and whisker plots, the line indicates the median (box hinge extends to the 25th and 75th percentile), and outlying points are identified as >1.5*IQR (interquartile range) from the hinge. Bar plots are displayed as mean±SD. Non-malignant samples are shown in blue, while tumor samples are shown in red.

Supplemental Figure 2

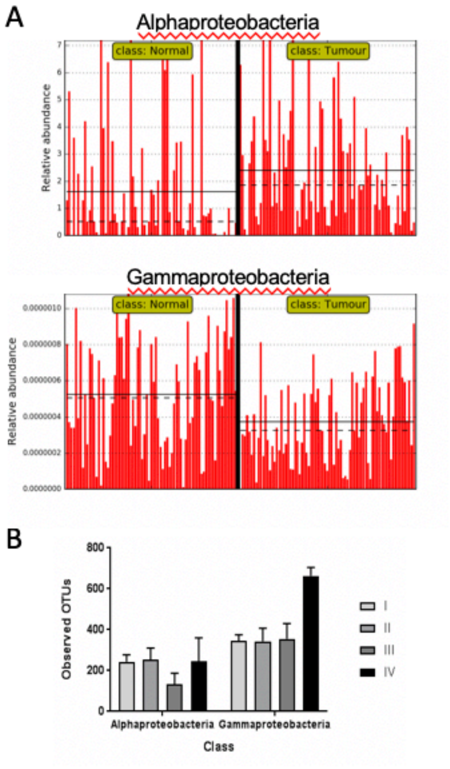

Supplemental Figure 2. Tumors are enriched in Alpha- and Gammaproteobacteria in a stage-independent manner. A) Relative abundance of Alphaproteobacteria and Gammaproteobacteria by class. B) Number of observed OTUs of each group by tumor stage.

Supplemental Figure 3

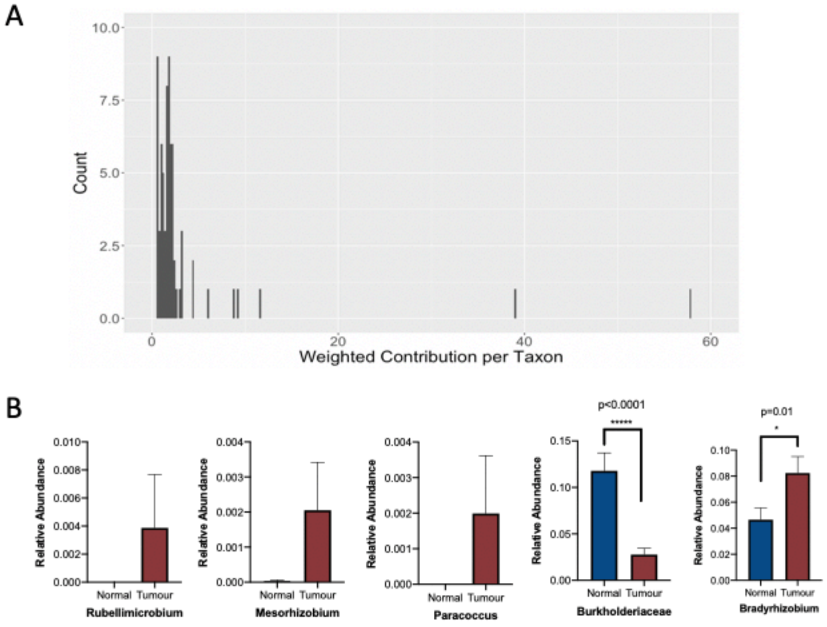

Supplemental Figure 3. Tumor samples are enriched for the taxons with the largest magnitude of predicted contribution to glycine betaine degradation. A) Histogram of weighted contribution per taxa to glycine betaine degradation pathway. Taxa with top 5 contribution to this pathway were assessed for relative abundance in tumor and paired non-malignant data. B) Relative abundance of the top 5 methionine-contributing taxa in tumor and paired normal tissue in the BCCRC dataset (see Figure 2B).

Supplemental Figure 4

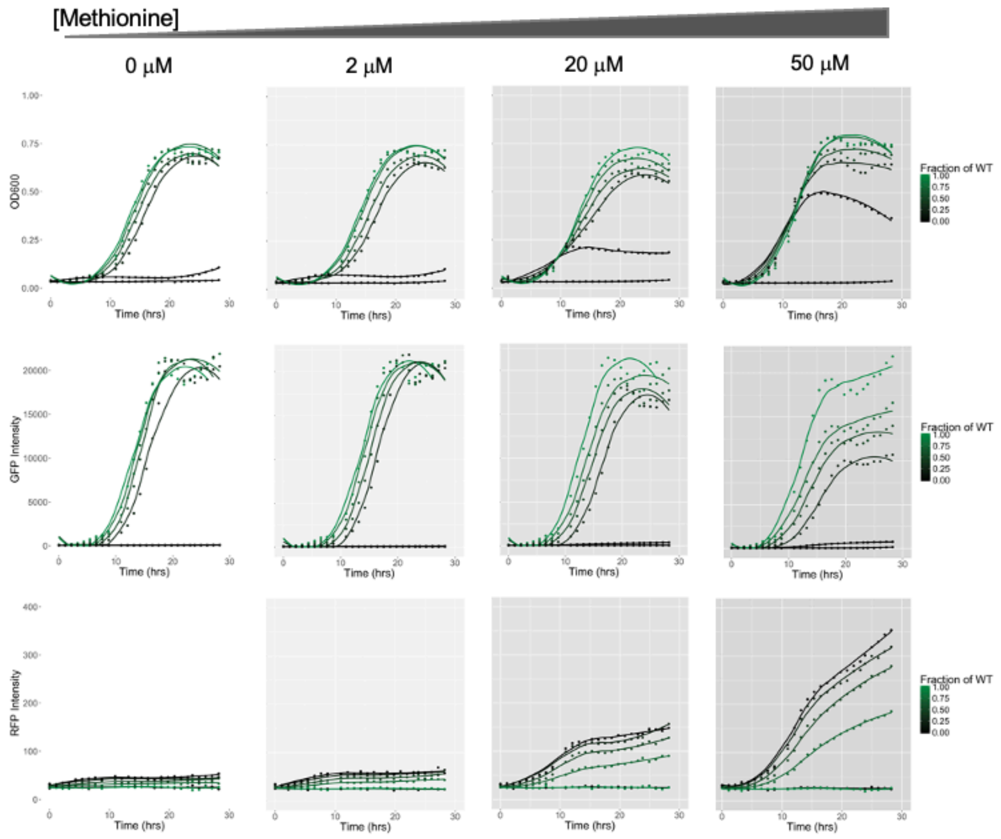

Supplemental Figure 4. Growth of methionine-producing and non-producing *E. coli* strains in varying concentrations of methionine at varying inoculation ratios. A) Bacterial growth at each inoculation fraction and methionine concentration is shown over time (to stationary phase, as assessed by OD600 plateau).

Supplemental Figure 5

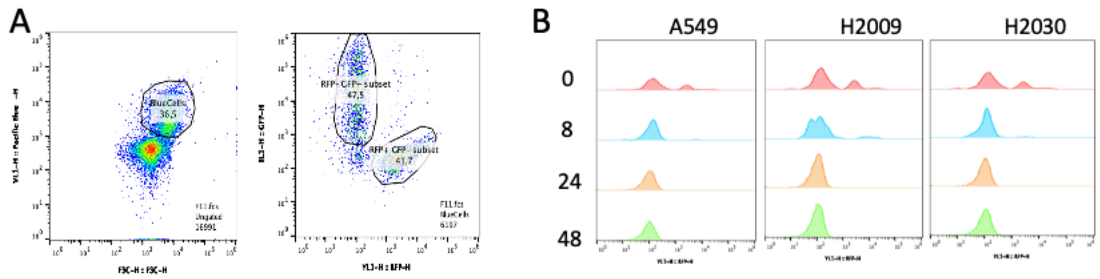

Supplemental Figure 5. Experimental design of bacterial-lung adenocarcinoma (LUAD) cell co-culture system. A) Gating strategy to assess GFP+ and RFP+ bacterial cell populations. B) Relative proportion of RFP+ cells present in cultures after exposure to LUAD cells (A549, H2009, and H2030) over time (8, 24, and 48hr intervals).

Supplemental Figure 6

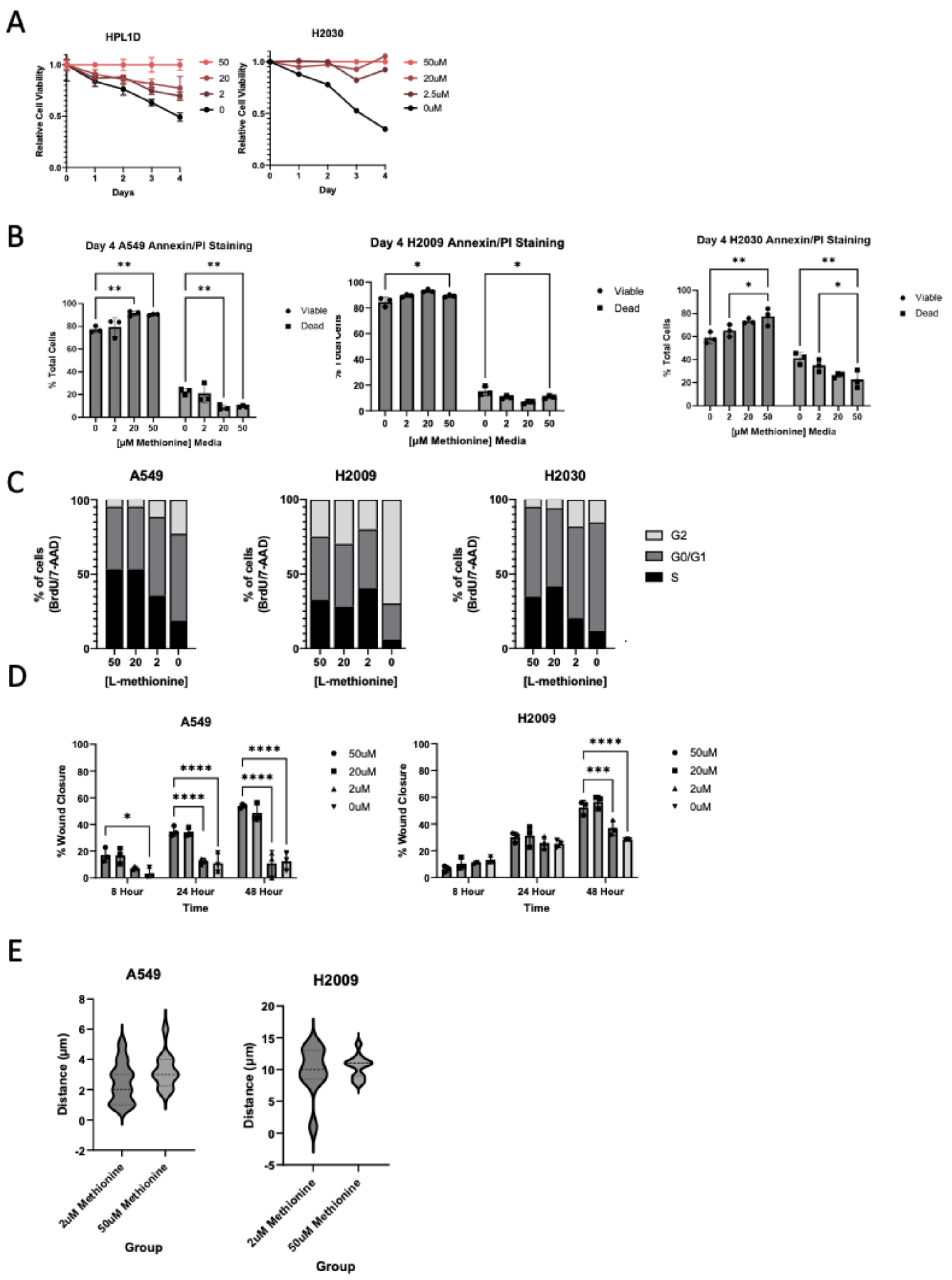

Supplemental Figure 6. Methionine deprivation results in decreased cell proliferation without little impact on motility in lung adenocarcinoma cell lines. A) Cell proliferation of transformed non-malignant lung tissue (HPL1D) and lung adenocarcinoma cell line (H2030) under decreasing levels of methionine was measured by AlamarBlue. B) Cell viability of LUAD cell lines were measured after 4 days of growth in different methionine concentrations by flow

cytometry. APC Anti-annexin V antibody and PI were used to differentiate viable cells with dead cells (Two-way ANOVA with Dunnett test, * $p<0.031$, ** $p<0.0021$) C) Cell cycle of A549, H2009, and H2030 grown in decreasing levels of methionine after 72 hours, measured using flow cytometry with anti-BrdU antibody and 7-AAD. D) Wound healing assay for A549 and H2009 cell lines. Wound images were acquired on hour 0, 8, 24, and 48 and ImageJ was used to quantify wound closure (Two-way ANOVA with Dunnett test, * $p<0.031$, *** $p<0.0002$, ****$p<0.0001$). E) Cell motility of A549 and H2009 using the Keyence Live Cell Imager. Cell tracker was used and total distance covered across 24 hours was plotted (student t-test, ns).

Supplemental Figure 7

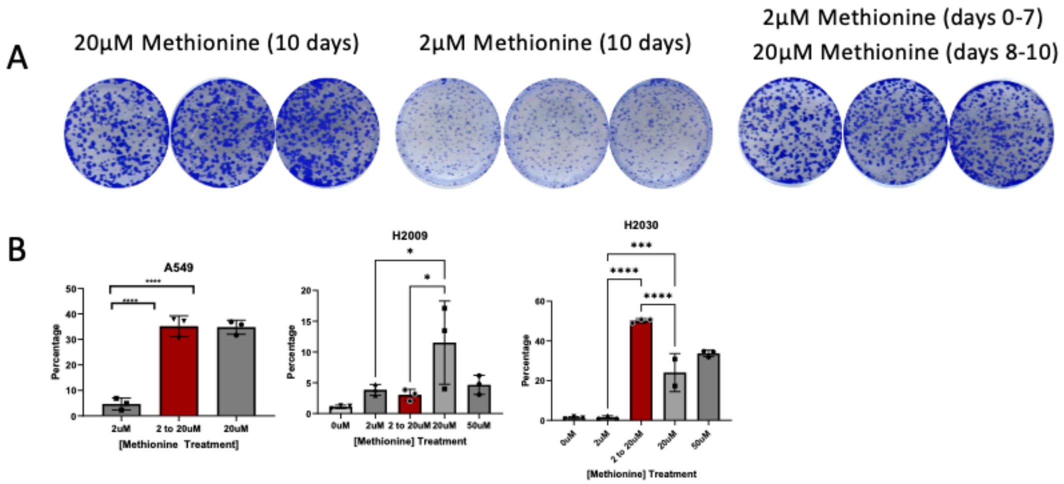

Supplemental Figure 7. LUAD phenotypes can be rescued either through reintroducing methionine or by introducing bacterial metabolites. A) Colony Forming Assay for A549 cells grown in either 20uM, 2uM, or rescued from 2uM to 20uM methionine containing media. Overall time in respective media is shown in paranthesis. B) Quantification of colony forming assay for A549, H2009, and H2030 with rescue experiment. Percentage of colony area was determined by ImageJ ColonyArea plugin (One-Way ANOVA with Sidak test, * $p<0.03$, *** $p<0.0002$, **** $p<0.0001$)

Supplementary Figure 8

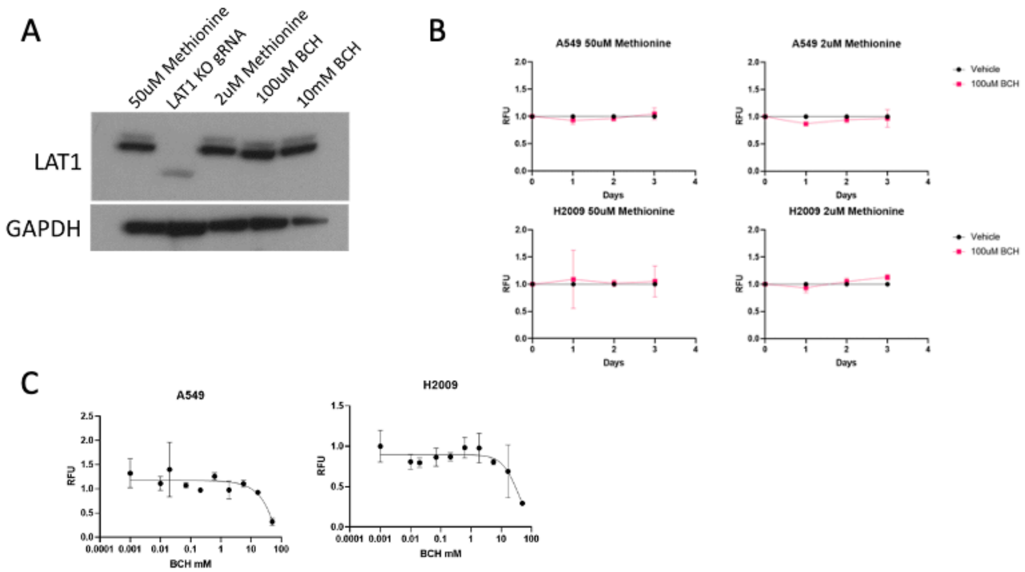

Supplementary Figure 8. LAT1 inhibition does not impact proliferation of LUAD. A) Western blot analysis of A549 cells in 50µM Methionine, LAT1 KO with guide RNA, 2µM Methionine, 100µM LAT1 inhibitor BCH, and 10mM BCH. B) alamarBlue reading of A549 and H2009 cell lines treated with PBS (Vehicle) or 100µM BCH in 50 or 2µM Methionine. E) alamarBlue reading of A549 and H2009 in increasing concentration of BCH treatment in 50µM Methionine.

Supplementary Figure 9

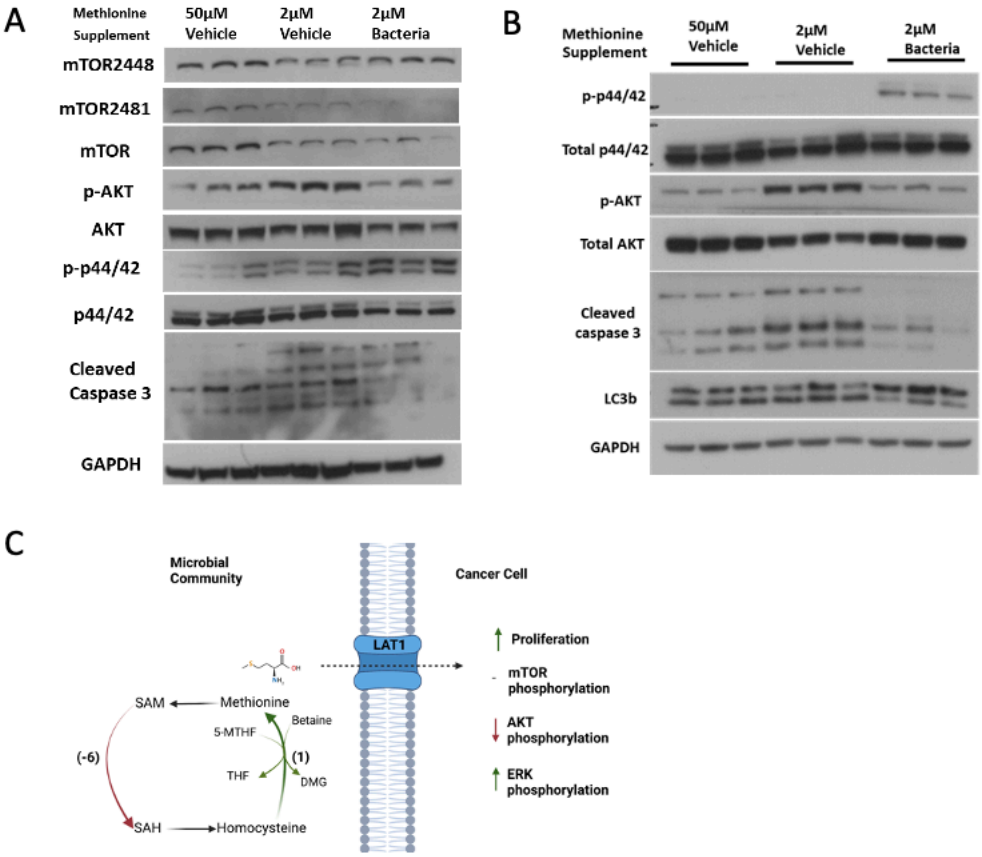

Supplementary Figure 9. Altered signaling in LUAD in the presence of bacteria. A) Western blot analysis of A549 and B) H2009 cells in 50μM methionine, 2μM methionine, or 2μM methionine with bacterially supplementation. C) Schematic representing overall signaling changes and phenotypic changes in response to bacterially supplemental media.
